# The Impact of the COVID-19 Pandemic on Health, Quality of Life and Intrafamilial Relations – A Population-Based Survey in Germany

**DOI:** 10.3389/fpsyg.2022.844057

**Published:** 2022-03-11

**Authors:** Stephanie Klein, Jörg M. Fegert, Alina Geprägs, Elmar Brähler, Vera Clemens

**Affiliations:** ^1^Hospital of Child and Adolescent Psychiatry/Psychotherapy, University of Ulm, Ulm, Germany; ^2^Department for Psychosomatic Medicine and Psychotherapy, University Medical Center of Johannes Gutenberg University of Mainz, Mainz, Germany

**Keywords:** COVID-19, pandemic, health, quality of life, intrafamilial relations, adverse childhood experiences

## Abstract

The occurrence of the novel severe acute respiratory syndrome coronavirus-2 (COVID-19) at the end of 2019 comes along with many challenges. Besides worry for one’s own health and the well-being of the family, all measures applied to limit the spread of the coronavirus affected daily life. School closures, economic shutdown and contact restrictions have led to high levels of stress. The impact on health and families has been widely discussed. However, population-based data are scarce. Here, we have assessed health, quality of life and intrafamilial relations depending on the COVID-19 pandemic. Using a three-step random-route approach, a population-based sample of 2,515 persons (52.6% female, average age of 50.3 years) was recruited during the second COVID-19 wave in Germany in winter 2020/21. While the majority of participants reported no change in their health status and the relationship with their partner and children, more than half of participants reported a decreased quality of life since the beginning of the pandemic. Female gender, age above 60 years, a low household income, not living with a partner and the experience of childhood adversity were associated with a higher risk for a worsening of health, quality of life and intrafamilial relations. These had already been well-established risk factors ahead of the pandemic. In order to avoid further increase of inequality in our society and more devastating impact of the pandemic on health and intrafamilial relations, low-level support and intervention programs are urgently needed.

## Introduction

After the occurrence of the novel severe acute respiratory syndrome coronavirus-2 (SARS-CoV-2) at the end of 2019, governments set up multiple restrictions to prevent the spread of the virus. These restrictions, including school closures, social distancing and economic shutdown helped to fight the increasing numbers of SARS-CoV-2 infections and death cases (Alfano and Ercolano, 2020). Isolation and social distancing, however, came along with an increase of mental health problems such as depression, acute stress or insomnia as discussed in reviews (Brooks et al., 2020; Holmes et al., 2020) and confirmed by multiple studies (Casagrande et al., 2020; Marroquín et al., 2020; Ford, 2021).

Once the World Health Organization (WHO) characterized the COVID-19 outbreak as a pandemic on March 11th 2020, many countries across the world faced several lockdowns which helped to limit the spread of the virus (Lau et al., 2020). People were ordered to work from home; schools, gastronomy, gyms and most shops were closed; and leisure activities were canceled. The first lockdown in Germany began on March 22, 2020 and encompassed contact restrictions, school and Kindergarten closures, orders to work from home and shutdown of public life with closing of shops (except supermarkets and drugstores) and service companies, ban on private gatherings and large events, and limitations in healthcare access, welfare and other support facilities. While the first lockdown ended *via* gradual relaxations – the first schools reopened on May 4th, the openings of schools and kindergartens stretched to the end of June 2020 -Germany has faced a second lockdown in winter 2020/2021 with again closures of most shops, service businesses, day care for children, schools switched to distance education (Bundeministerium für Gesundheit, 2022). Since the end of 2021, Germany is again facing rising numbers of SARS-CoV-2 infections. In total, to date more than 10 million SARS-CoV-2 infections have been reported in Germany and more than 100,000 persons have died with COVID-19 (Robert Koch-Institut, 2022).

Due to the above named restrictions people, especially families, have faced new challenges: no separation of work from leisure time, sharing limited space and electronic devices with many family members, working from home and taking care of the children, home-schooling and social distancing.

These new and unknown circumstances, the medical threat of the virus itself and the economic insecurities, which came along with restrictions led to increasing stress and mental health problems, while many opportunities to cope with these such as meeting family and friends and out-of-home leisure time activities were also restricted (Flaxman et al., 2020).

Closures of day care facilities for children and school closures were shown to affect particularly women in Germany, who carried the main burden of child care while facing greater income losses than men (Kohlrausch and Aline, 2020). Due to the first lockdown, in 2020, the gross domestic product in Germany declined sharply (Statistisches Bundesamt, 2022). This economic burden has been distributed unequally in the German population, hitting in particular persons with preexisting low income and in precarious working conditions (Hövermann, 2020). Thus, females and people with lower household income may be at particular risk to suffer from long-term sequelae resulting from the pandemic. Female gender was shown to be directly associated with negative beliefs about COVID-19’s consequences, and associated affects and beliefs (Ceccato et al., 2021). Importantly, female gender and a low income are risk factors for the development of post-traumatic stress disorder (PTSD) in general (Tang et al., 2017) and in the context of the COVID-19 pandemic (Di Crosta et al., 2020). Precarious working conditions were not only shown to be associated with poorer health and lower life satisfaction (Cheng and Chan, 2008), but also higher propensity to reduce short-term consumptions and greater perceived unaffordability of long-term life projects such as buying a house during the pandemic, potentially further affecting life quality (Chirumbolo et al., 2021). The experience of adverse childhood experiences (ACEs) were shown to be associated with dysfunctional coping strategies (Clemens et al., 2021a; Köhler-Dauner et al., 2021) and thus a higher risk for intrafamiliar problems (Clemens et al., 2021a,b; Sachser et al., 2021).

While there are reviews, reports and studies about the negative impact of the COVID-19 pandemic on family well-being (Prime et al., 2020), partner relationship (Sachser et al., 2021), children and adolescents (Fegert et al., 2020; Guessoum et al., 2020) and mental health in general (Hossain et al., 2020), there is a lack of studies assessing risk factors for coping with the COVID-19 pandemic in the general public. Studies in representative samples are necessary to assess valid, population-based risk factors that overcome bias in convenience samples. Previously, we have shown that during the first lockdown in Germany, fewer mental health problems were reported by a population-based sample compared to before lockdown, while no significant differences were seen in partnership quality (Sachser et al., 2021). These associations were age- and income-dependent. However, the pandemic has not ended after the first lockdown. Germany, as well as several other countries worldwide, has faced second and third infection waves and further lockdowns. Currently, a fourth wave is hitting multiple regions of the world. Literature points toward an increasing load of social and health problems with increasing duration of the COVID-19 pandemic and associated restrictions in Germany (Ahrens et al., 2021; Moradian et al., 2021). However, population-based representative data are missing.

Therefore, here, we aimed to assess the change of individual health and life quality as well as intrafamilial relationships during the second wave of the COVID-19 pandemic in winter 2020/21 in a representative sample of the German population. Moreover, we analyzed the impact of population-based risk factors, including gender, age, household income and the experience of childhood adversity. Based on the current literature, we hypothesized an overall decrease of quality of life in the general population. As risk factors, we hypothesized female gender, lower household income, and the experience of childhood adversity.

## Materials and Methods

### Study Design

A representative sample of the German population was obtained by USUMA, an opinion research institute based in Berlin, Germany. In a first selection step, a systematic area sampling (ADM F2F Sampling Frame), based on the municipal classification of the Federal Republic of Germany, covering the inhabited territory of Germany, was used. On this basis, around 53,000 segments were delimited electronically, containing at least 350 and on average 700 private households in each area. These areas were firstly layered regionally according to districts into around 1,500 layers and secondly divided into 128 strictly disjunct “networks.” In the next step, one such network served as sampling frame, each network contained 258 single sample points proportionate to the distribution of private households in Germany. Then, in a second selection step, private households were systematically selected at each sample point using a random route procedure. Based on strict routing instructions, households of every third residence in randomly assigned streets selected and invited to participate in the study. In a third and last selection step, the target person in the respective household was randomly chosen within those households using a *Kish-Grid* selection. The survey took place from December 14th 2020 – March 7th 2021 and thereby took place during the second wave of COVID-19 and lockdown in Germany. Participants had to be at least 16 years old and have sufficient German language skills to participate.

Persons who agreed to participate were given information about the study and provided informed consent. Firstly, socio-demographic information was obtained in an interview-format by the research staff. Then, the researcher handed out the questionnaire and a sealable envelope. During completion of the questionnaire, the researcher remained nearby in case the participants needed further information or left the household based on the participants wishes. After completion, the questionnaires were handed back in the sealed envelope. Questionnaire data were linked to the respondent’s demographic data, but did not contain name, address, or any other identifying information.

Of 5,913 initially contacted households, 2,519 people filled out the survey (response rate: 42.6%). The main reasons for non-participation were refusal of the selected household to provide information (23.8%), failure to contact persons in the household after four attempts (14.1%) and refusal of the target person to participate (13.5%).

### Ethics

The study was conducted in accordance with the Declaration of Helsinki and was approved by the Ethics Committee of the Medical Department of the University of Leipzig (AZ-474/20-ek).

### Measures

Socio-demographic questions encompassed age, gender, income, children below the age of 16 and living with a partner.

Regarding the general condition during the pandemic compared to the time before the pandemic, participants were asked to describe their current health status, their relationship with their partner, the relationship to their children and their current quality of life in comparison to before the pandemic. In detail, the questions used were “Compared to before the COVID-19 pandemic, how would you describe your current general health?,” “Compared to before the COVID-19 pandemic, how would you describe your current quality of life?,” “Compared to before the COVID-19 pandemic, how would you describe the relationship with your partner at present?,” “Compared to before the COVID-19 pandemic, how would you describe your relationship to your children at present?.” Possible answers were “Currently much better than before,” “Currently somewhat better than before,” “Currently about the same,” “Currently somewhat worse than before,” and “Currently much worse than before.” While for descriptive analyses all five possible answers were used, for regression analyses answers were summarized into the categories “better,” “equal,” and “worse.”

Adverse childhood experiences were assessed using the German version of the Adverse Childhood Questionnaire (Felitti et al., 1998), a standard tool for retrospective assessment of ACEs with satisfactory reliability [Cronbachs α = 0.76 (Wingenfeld et al., 2011)]. The questionnaire captures five forms of child maltreatment: physical abuse; emotional abuse; sexual abuse; physical neglect; and emotional neglect - and five forms of household dysfunctions: substance abuse and mental illness of a family member; intimate partner violence between parents; incarceration of a family member and disappearance of a parent through divorce, death or other reason. A sum score between “0” (no experiences of any ACE) and “10” (having experienced all 10 assessed forms of ACEs) can be calculated (Felitti et al., 1998) and was used in this sum form in the here described analyses.

### Data Analysis

All statistical analyses were performed with SPSS version 27. Prevalence rates were performed by descriptive analyses. In order to illustrate the possible risk factors for worse coping with the COVID-19 Pandemic we examined our data on the variables gender, equalized household income (<1,000€ vs. 1,000–2,000€ vs. 2,000–3,000€ vs. >3,000€) and number of ACEs (0 vs. 1–3 vs. ≥4).

Multinomial Regression analyses were performed to identify factors associated with successful coping of pandemic-related challenges. Sociodemographic variables (gender, age, equalized household income, living with a partner, children under 16 years in the household) and the number of experienced forms of ACEs were included as determinants while COVID-19-related variables (differences in current health status, quality of life and relationship with partner and children compared to the time before the COVID-19 pandemic) served as outcomes. Number of ACEs, age and household income were categorized into each three groups. For ACEs these groups were: 0 vs. 1–3 vs. 4 and more ACEs, age was separated into groups of 16 to 34 vs. 35 to 59 vs. 60 years and older and household income was grouped into under 1,000€ vs. 1,000–3,000€ vs. 3,000€ and above. Due to the small number (*n* = 4) of respondents describing themselves as non-binary/third gender in the sample, these persons were excluded from analyses.

## Results

### Participants

A total of 2,519 participants completed the survey. 1322 (52.6%) of them were female, *n* = 4 were non-binary/third gender. Mean age of the sample was 50.51 (±18.05) years for females and 50.14 (±17.72) years for males (age range 16–96). Half of the participants lived with a partner (f: *N* = 737, 56.6%; m: *N* = 729, 62.1%). The majority held the German citizenship (f: *N* = 1,271, 96.4%; m: *N* = 1,151, 96.5%). Demographic information is displayed in Table 1.

**TABLE 1:** Sample characteristics.

	Total 2515	Female (%) 1322 (52.6)	Male (%) 1193 (47.4)
Age, mean (SD), years	50.33 (18.05)	50.51 (18.34)	50.14 (17.72)
16–34 years, *n* (%)	591 (23.5)	299 (22.6)	292 (24.5)
45–59 years, *n* (%)	1086 (43.2)	580 (43.9)	506 (42.4)
≥60 years, *n* (%)	838 (33.3)	443 (33.5)	395 (33.1)
German citizenship, *n* (%)	2422 (96.4)	1271 (96.4)	1151 (96.5)
Equalized disposable household income, *n* (%)			
<1,000 €	255 (10.4)	138 (10.8)	117 (10.0)
1,000–3,000 €	1825 (74.5)	972 (75.9)	853 (72.8)
>3,000 €	372 (15.2)	171 (13.3)	201 (17.2)
Living with partner, *n* (%)	1466 (59.2)	737 (56.6)	729 (62.1)
Children < 16 years in household, *n* (%)	438 (17.4)	266 (20.1)	172 (14.4)
Number of experienced ACEs, *n* (%)			
0	1678 (67.4)	879 (67.2)	799 (67.7)
1–3	574 (23.1)	288 (22.0)	286 (24.2)
4–10	238 (9.6)	142 (10.8)	96 (8.1)

*Presented as number (n) or mean value (M) and standard variation (SD) and (%).*

### Change of Health, Quality of Life and Intrafamilial Relations During the Pandemic

**FIGURE 1:**
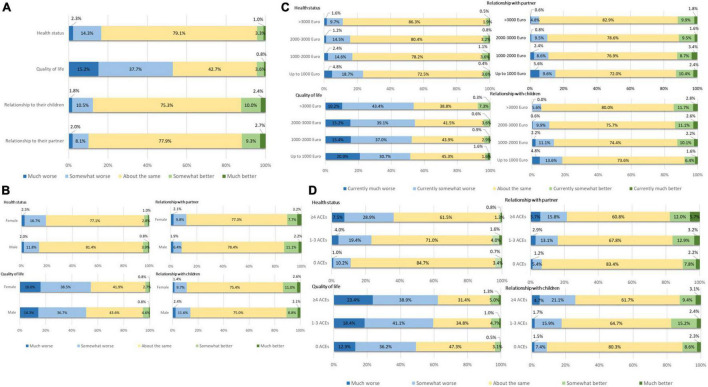
Impact of COVID-19 and associated measures on health, quality of life and intrafamilial relations. **(A)** General impact. Impact in dependence of gender **(B)**, household income **(C)**, and adverse childhood experiences **(D)**.

Focusing on the total sample, the majority of participants stated no difference regarding their health status or the relationship with their partner and their children during the second wave compared to before the pandemic. About the same proportion of participants reported a worsening such as an improvement of the relationship with their partner and their children. Focusing on health, about one quarter reported a worsening of the general health status and only 4% an improvement of the general health status since the beginning of the pandemic. Regarding quality of life, the majority of participants reported a worsening compared to the time before the pandemic (see Figure 1A).

Subgroup analyses revealed that a worsening of general health was more likely in female participants (χ^2^ = 15.50, *p* = 0.004), participants with lower equalized household income (χ^2^ = 141.26, *p* < 0.001) and in participants with the experience of childhood adversity (χ^2^ = 29.23, *p* = 0.004). Focusing on quality of life, the experience of ACEs was associated with higher rates of worsening since the beginning of the pandemic (χ^2^ = 53.73, *p* < 0.001). Participants with lower income were more likely to report a strong worsening of quality of life and less likely to report an improvement compared to participants with higher income (χ^2^ = 42.91, *p* < 0.001). Women reported more often a worsening of the relationship with their partner compared to men (χ^2^ = 13.32, *p* = 0.010). Moreover, the experience of ACEs (χ^2^ = 82.33, *p* < 0.001) and a lower income (χ^2^ = 29.56, *p* = 0.003) were associated more frequently with a worsening of the relationship with the partner since the beginning of the pandemic. Participants with a lower income (χ^2^ = 22.82, *p* = 0.03) and the experience of childhood adversity (χ^2^ = 57.03, *p* < 0.001) were more likely to report a worsening and less likely to report an improvement of the relation to their children compared to the time before the pandemic (see Figures 1B–D).

### Factors Associated With a Worse Health State During the Pandemic

Results of the regression analyses revealed that age higher than 60 years (OR = 3.833, *p* < 0.001), the experience of 4–10 ACEs (OR = 9.431, *p* < 0.001) and not living with the partner (OR = 1.156, *p* < 0.05) were associated with a higher risk for a currently worse state of health compared to before the pandemic. Male gender (OR = 0.519, *p* < 0.05) predicted a better health status in comparison to before the pandemic.

### Change of the Relationship With the Partner During the Pandemic

Higher risk for a deterioration of the relationship with their partner was associated with score of 4–10 ACEs (OR = 1.831, *p* < 0.05) and a household income between 1,000–3,000€ (OR = 2.022, *p* < 0.05), while male gender (OR = 0.565, *p* < 0.01) was associated with a lower risk to report a worsening of the relationship with the partner during the pandemic (for details see Table 2).

**TABLE 2:** Factors associated to equally and worse rated health and intrafamilial relations compared to improvements since the beginning of the pandemic.

Compared to the time before the pandemic:	Current health state is equal			Relationship to partner is equal			Relationship to children is equal			Current quality of life is equal		
	Odds ratio^1^	95% CI	*p*-value	Odds ratio^1^	95% CI	*p*-value	Odds ratio^1^	95% CI	*p*-value	Odds ratio^1^	95% CI	*p*-value
Male Gender	0.790	0.522; 1.197	0.266	0.782	0.577; 1.061	0.114	1.244	0.874; 1.772	0.225	0.627	0.412; 0.954	0.03
Age in years 35–59	0.950	0.579; 1.560	0.839	1.194	0.826; 1.728	0.346	0.558	0.325; 0.959	0.035	1.431	0.885; 2.313	0.144
>60	2.295	1.211; 4.347	0.011	1.899	1.206; 2.989	0.006	0.944	0.476; 1.874	0.870	3.327	1.740; 6.362	<0.001
ACE score 1–3 ACEs	0.589	0.376; 0.924	0.021	0.547	0.385; 0.777	0.001	0.498	0.338; 0.734	0.000	0.429	0.268; 0.686	<0.001
4–10 ACEs	2.192	0.675; 7.120	0.192	0.416	0.257; 0.674	0.000	0.613	0.339; 1.108	0.105	0.268	0.153; 0.548	<0.001
Household income <1000€	0.315	0.118; 0.844	0.022	0.834	0.436; 1.598	0.585	1.750	0.761; 4.028	0.188	2.138	0.851; 5.370	0.106
1000–3000 €	0.365	0.178; 0.749	0.006	0.958	0.647; 1.418	0.831	1.155	0.711; 1.876	0.560	1.740	1.049; 2.886	0.320
Not living with a partner	2.423	1.472; 3.989	<0.001	–	–		1.087	0.695; 1.699	0.716	3.740	2.084; 6.711	<0.001
No children living in the household	0.637	0.366; 1.108	0.110	1.026	0.702; 1.497	0.896	1.898	1.236; 2.914	0.003	1.083	0.644; 1.823	0.763

**Compared to the time before the pandemic:**	**Current health state is worse**			**Relationship to partner is worse**			**Relationship to children is worse**			**Current quality of life is worse**		
	**Odds ratio**	**95% CI**	***p*-value**	**Odds ratio**	**95% CI**	***p*-value**	**Odds ratio**	**95% CI**	***p*-value**	**Odds ratio**	**95% CI**	***p*-value**

Male Gender	0.519	0.328; 0.819	0.005	0.565	0.371; 0.862	0.008	1.738	1.087; 2.780	0.021	0.556	0.368; 0.841	0.005
Age in years 35–59	1.259	0.716; 2.211	0.424	1.543	0.916; 2.599	0.103	1.212	0.514; 2.855	0.661	1.311	0.819; 2.099	0.260
>60	3.833	1.909; 7.697	0.000	1.342	0.706; 2.550	0.369	3.453	1.263; 9.436	0.016	2.781	1.465; 5.282	0.002
ACE score 1–3 ACEs	1.601	0.978; 2.622	0.061	1.643	1.026; 2.631	0.039	1.294	0.777; 2.155	0.323	0.721	0.455; 1.140	0.162
4–10 ACEs	9.431	2.852; 31.186	<0.001	1.831	1.005; 3.338	0.048	2.262	1.123; 4.557	0.022	0.569	0.308; 1.051	0.072
Household income < 1000 €	0.655	0.226; 1.869	0.436	2.431	0.997; 5.924	0.051	4.465	1.471; 13.554 1.075	0.008	1.591	0.641; 3.951 0.91	0.317
1000–3000 €	0.560	0.254; 1.233	0.150	2.022	1.086; 3.764	0.026	2.389	; 5.309	0.033	1.494	1; 2.449	0.112
Not living with a partner	1.156	1.156; 3.393	0.013	–	–		1.376	0.788; 2.402	0.262	3.496	1.957; 6.245	<0.001
No children living in the household	0.726	0.390; 1.352	0.313	1.201	0.716; 2.015	0.488	1.333	0.709; 2.503	0.372	1.045	0.628; 1.740	0.864

*^1^An OR > 1 corresponds to a higher probability of reporting that the respective outcome is equal compared to being better than before the pandemic.*

*Comparison categories from top to bottom: gender female, Age: 16–34 years, ACE score: 0 ACEs, Household income > 3000€, Living with a partner, Children living in the household.*

### Factors Associated to a Change in the Relationship With the Children During the Pandemic

A decrease in the quality of the relationship with their children was seen in participants with male gender (OR = 1.738, *p* < 0.05), age above 60 years (OR = 3.453, *p* < 0.05), participants who reported the experience of 4–10 ACEs (OR = 2.262, *p* < 0.05) and household income below 1,000€ (OR = 4.465, *p* < 0.01) (for details see Table 2).

### Factors Associated to a Change of the Quality of Life

Male gender (OR = 0.556, *p* < 0.01) was associated with a lower risk for a decreased quality of life during the pandemic, corresponding to a higher risk for a decreased quality of life in females. Age above 60 years (OR = 2.781, *p* < 0.01) and not living together with a partner (OR = 3.496, *p* < 0.001) came along with a higher risk for a decrease of quality of life (for details see Table 2).

## Discussion

To the best of our knowledge, this is among the first population-based studies in Germany assessing health, quality of life and intrafamilial relations during the second wave of the COVID-19 pandemic.

The majority of participants stated no difference, neither regarding their health status, nor regarding the relationship with their partner and their children during the second wave compared to before the pandemic. This is in line with a population-based study in Germany during the first lockdown (Sachser et al., 2021) where an improvement of mental health was described by a comparable sample and no differences in the relation to the partner. However, about one quarter of participants reported a worsening of the general health status. The reported worsening in health in our study contrasts the sample described above (Sachser et al., 2021). The current study was conducted at a later point of time of the course of the pandemic during the second wave of COVID-19 in Germany, and thus contrasts with the findings of the study by Sachser et al. (2021) which was based on the first lockdown period. Health problems were shown to accumulate with increasing length of the COVID-19 pandemic (Ahrens et al., 2021; Moradian et al., 2021). Moreover, in our study, not only mental health but also general health was assessed. Our data are in line with the results of a representative longitudinal sample from the United Kingdom where about two fifths of participants showed severely increased risk for distress during the pandemic (Ellwardt and Präg, 2021). Regarding quality of life, most participants reported a worsening compared to the time before the pandemic. This can be easily explained by the pandemic-associated restrictions in daily life and is in line with literature (Ferreira et al., 2021; Ravens-Sieberer et al., 2021). Besides everyday restrictions, economic pressure may have had a significant impact on the general worsening of quality of life as well. Due to the pandemic, Germany has faced a financial crisis (Statistisches Bundesamt, 2022), which has hit in particular persons with preexisting low income and in precarious working conditions (Hövermann, 2020). As economic hardship and job insecurity are well known to reduce life quality (Cheng and Chan, 2008), this may be another relevant factor for the seen overall decrease in life quality.

Several factors critically influenced the change of the assessed factors including gender, age, household income, living with a partner and importantly the experience of adversity during childhood and/or adolescence. Women are generally at higher risk for mental health problems such as depression or anxiety (Boyd et al., 2015; Dreger et al., 2016). During the COVID-19 pandemic, this gender difference even exacerbated (Covid-19 Mental Disorders Collaborators, 2021). Women were shown to have higher levels of anxiety and depression (Benke et al., 2020; Abreu et al., 2021; Ferreira et al., 2021) and a lower quality of life (Teotônio et al., 2020; Ferreira et al., 2021) compared to men during the pandemic. In line with these results, our data show enhanced risk for a decreased general health status, and furthermore enhanced risk for a decrease of quality of life and relationship with their partner compared to men. These findings are not surprising. Women have carried the main burden of increased caring responsibility due to school closures (United Nations, 2020). They were at higher risk for financial loss due to COVID-19 (Wenham et al., 2020) and domestic violence (Piquero et al., 2021). Moreover, female gender is a risk factors for the development of PTSD in general (Tang et al., 2017) and in the context of the COVID-19 pandemic (Di Crosta et al., 2020). PTSD is discussed as one consequence of the pandemic in general (Di Crosta et al., 2020), and elevated post-traumatic stress symptoms were seen in COVID-19 survivors (Tu et al., 2021).

Focusing on age, people above 60 had the most severe symptoms and a higher risk of mortality from SARS-Cov2 infection (Parohan et al., 2020; Pollard et al., 2020). Literature points toward a higher risk for being socially and emotionally lonely (van Tilburg et al., 2021). In our study, participants above the age of 60 had two to four times higher risk for a decrease in general health status, quality of life and relationship with their children compared to participants aged 16–34. These findings are in line with other studies, showing higher levels of anxiety, poorer sleep quality, stress and depression in the older population (Lee et al., 2020; Sepúlveda-Loyola et al., 2020). In contrast to our findings, there is literature pointing out that not the elderly but young people are at higher risk for mental health problems during the COVID-19 pandemic, such as depression, anxiety or stress (Huang and Zhao, 2020; Pieh et al., 2020; Covid-19 Mental Disorders Collaborators, 2021). These results could be explained by more uncertain working conditions and more financial problems for younger people during lockdown (Pieh et al., 2020). Moreover, young people have been particularly hit by pandemic-associated restrictions, reduction of social contacts and have experienced greater perceived changes in life. These factors were shown to be associated with higher mental health impairments (Benke et al., 2020). However, in our population-based sample, after adjustment for household income as confounder, the decrease in general health status, quality of life and relationship with their children in the elderly remained significant. The generally higher need for medical care in the older population may have led to a higher impact of reduction of health care in the elderly. COVID-19-associated contact restrictions have affected older population in particular (van Tilburg et al., 2021). In Germany, the majority of children move out of their parents’ home after reaching adulthood. Consequently, contact for older people with their children was restricted, which may have influenced their relationship.

The impact of low income and economic hardship on health, quality of life and intrafamilial relationships has been shown numerously in pre-pandemic times (Schneider et al., 2016, 2017). Our data reveal a significant impact of household income on coping with COVID-19-associated challenges. A low income was associated with a nearly five times increased risk for a worse relationship with their children compared to participants with a high income. These findings correspond with other studies reporting significant parenting-related exhaustion, a decrease of parental satisfaction with work-family balance, and increased risk for physical and psychological violence depending on economic hardship and income loss (Brown et al., 2020; Craig and Churchill, 2020; Clemens et al., 2021b). Importantly, our data do not point toward a general worsening of intrafamilial relationships during the pandemic. This is interesting, as increased stress levels, the effect of working from home while taking care for children and homeschooling has been reported several times (Fegert et al., 2020; Calvano et al., 2021). However, based on our data, this seems to have only affected the relationship with children if other factors, such as financial stress, occur.

Our study underlines the significance of the experience of childhood adversity on coping with pandemic-associated challenges. Participants who had experienced four or more ACEs were about two times more likely to report a worsening of their relationship with their partner or children. There was a tenfold increase in the risk of worsening of the current health status, Even in less stressful times, people who had experienced a high number of ACEs have an elevated risk for mental health problems like depression, anxiety, substance use disorder and a significant reduction in quality of life (Norman et al., 2012; Witt et al., 2019), as well as for somatic health problems including severe obesity, ischemic heart disease, diabetes or the occurrence of any kind of cancer (Felitti et al., 1998; Clemens et al., 2018). ACEs have been shown numerously to affect intrafamilial relations negatively (Dixon et al., 2005; Clemens et al., 2020). A higher stress-vulnerability and a decrease of emotion regulation (Hein and Monk, 2017; Duffy et al., 2018) was shown for people who have experienced ACEs, affecting coping of stressful situations. Focusing on the COVID-19 pandemic, our results are in line with literature reporting significantly higher level of anxiety, depression and PTSD in Chinese students with childhood adversity during the pandemic (Di Crosta et al., 2020). Our own research points toward an increased risk for burn-out and potentially harmful parenting methods during the pandemic depending on the experience of childhood adversity (Clemens et al., 2021a,b).

## Conclusion

Taken together, our data show impressively the impact of the pandemic on health, quality of life and intrafamilial relations in the German population. Income, older age, female gender and the experience of childhood adversity comprise significant population-based risk factors for coping with the pandemic. These had already been well-established risk factors ahead of the pandemic. Thus, people who are at high risk for health and social problems in more normal circumstances may be hit by the pandemic particularly. Furthermore, during the pandemic and lockdown measures, health institutions like therapy or social work supporting offers were restricted. Problems were less likely to be treated adequately which increases the likelihood of long-term health consequences and chronic manifestations. Literature points toward an accumulative effect of the pandemic and associated measures on health and social life of the society when the situation persists. As the current pandemic is far from being over, low-level support and intervention programs are needed to avoid further inequality in our society and intensification of the devastating impact of the pandemic on health and intrafamilial relations – in the general public, but predominantly in persons who are already particularly vulnerable.

## Data Availability Statement

The data supporting the conclusions of this article will be made available on reasonable request.

## Ethics Statement

The studies involving human participants were reviewed and approved by the Ethics Committee of the Medical Department of the University of Leipzig. Written informed consent to participate in this study was provided by the participants’ legal guardian/next of kin.

## Author Contributions

SK, AG, and VC interpreted the data and wrote the manuscript. EB supported recruitment of the sample. JF and VC conceptualized the survey and supervised data analyses. All authors read and approved the final manuscript.

## Conflict of Interest

JF has received research funding from the EU, DFG (German Research Foundation), BMG (Federal Ministry of Health), BMBF (Federal Ministry of Education and Research), BMFSFJ (Federal Ministry of Family, Senior Citizens, Women and Youth), G-BA Innovationsfonds, several state ministries, State Foundation Baden-Württemberg, Volkswagen Foundation, Porticus Foundation, Diocese of Rottenburg-Stuttgart. Moreover, he received travel grants, honoraria and sponsoring for conferences and medical educational purposes from APK, Deutschlandfunk, DFG, DJI, DKSB, Infectopharm, med update, UNICEF, several universities, professional associations, political foundations, and German federal and state ministries during the last 5 years. JF holds no stocks of pharmaceutical companies. The remaining authors declare that the research was conducted in the absence of any commercial or financial relationships that could be construed as a potential conflict of interest.

## Publisher’s Note

All claims expressed in this article are solely those of the authors and do not necessarily represent those of their affiliated organizations, or those of the publisher, the editors and the reviewers. Any product that may be evaluated in this article, or claim that may be made by its manufacturer, is not guaranteed or endorsed by the publisher.
